# Are you understanding what I am saying? The critical importance of communication competency in epidemiology

**DOI:** 10.3389/fpubh.2025.1533393

**Published:** 2025-01-31

**Authors:** Alison G. Abraham, WayWay M. Hlaing

**Affiliations:** ^1^Department of Epidemiology, University of Colorado, Anschutz Medical Campus, Aurora, CO, United States; ^2^Department of Ophthalmology, University of Colorado, Anschutz Medical Campus, Aurora, CO, United States; ^3^Division of Epidemiology and Population Health Sciences, Department of Public Health Sciences, Miller School of Medicine, University of Miami, Miami, FL, United States

**Keywords:** communication, curriculum, epidemiology, misinformation, training

## Abstract

There are myriad examples of poor communication by public health scientists and researchers that have resulted in lasting harm to individuals, communities, the field of epidemiology, and the broader field of public health. These examples underscore that science messages hinge not only on their merit alone but also on how effectively we communicate them. Here, we highlight the strong consensus in the epidemiology educational literature that epidemiology students should be trained to communicate effectively, specifically with the general public. This allows the public access to critical information that could affect their well-being. Most epidemiology programs in academia do not focus on the skills needed to translate scientific evidence and its uncertainty into a comprehensible and culturally appropriate message to the diverse public composed of varying race/ethnicities as well as varying health and numerical literacy levels. We provide guidance on which specific communication skills may be most important for epidemiologists facing the growing health misinformation and disinformation epidemic. We also describe what a communication-focused curriculum might look like, given that communication skills cannot be learned solely through traditional coursework. Lastly, we address barriers that have prevented communication skills from being meaningfully incorporated in epidemiology curricula.

## Introduction

In June of 2002, one arm of the Women’s Health Initiative (WHI) trial, a randomized clinical trial of hormone replacement therapy (HRT) in 160,000 postmenopausal women, was halted. The WHI steering committee organized a major press conference in July to announce the findings of the study. The acting director of the WHI told the press that the trial results indicated HRT conferred a small but statistically significant increased risk of cardiac events, strokes and clots, as well as an increased risk of breast cancer. “The adverse effects outweigh and outnumber the benefits,” he stated. In the weeks to come, this message would be disseminated and amplified in a way that was later described as “an exercise in poor communication that would have profound repercussions for decades to come” (1). Alarming statistics were thrown at lay audiences, presenting accurate but misleading relative risks of outcomes like heart disease and breast cancer associated with HRT, without the accompanying context of the small absolute risks of those events. Limitations of the study, like its over-recruitment of women 60 and older and relative paucity of healthier women in their 50s, were not mentioned. In fact, the study was described as broadly applicable. Tradeoffs with benefits such as bone health were not emphasized or well communicated in the messaging despite an estimated 40% lifetime risk of osteoporotic fracture for White American women aged 50 years (2) and WHI data available at the time showing women on HRT experiencing 24 percent fewer fractures (1, 3).

Still today, despite overwhelming evidence suggesting the benefits of HRT for women in their 40s and 50s and heroic efforts by researchers in the field to counter perceptions (4, 5), HRT remains under-prescribed (6), and millions of menopausal women needlessly suffer hot flashes that disrupt sleep, mood changes, difficulty concentrating, and impairment of short-term memory (7).

The history of HRT is a case study of the dire and lasting consequences of a public health workforce untrained in the art and science of communication. It is hardly the only example. Ironically the earlier 1995 Pill Scare in England also resulted from poor communication over risks associated with the use of hormones by women. In this case, at issue was the risk of venous thromboembolism in women taking oral contraceptive pills with third generation progestins. The Committee on Safety of Medicines sent a warning to British doctors based on three yet-to-be published studies cautioning use because of a suggested doubling of risk of venous thromboembolism, but neglected to communicate the rarity of the event (the absolute risk), which occurs in an estimated 2 out of every 7,000 women taking such oral contraceptives (8). Nor were the many benefits of oral contraceptives communicated, or the limitations of the epidemiologic evidence. Following the media attention about the warning, thousands of women discontinued the use of the pill resulting in a substantial increase in the number of unintended pregnancies and abortions, both of which involve higher risks of venous thromboembolism than oral contraceptive use (9).

Finally, we need to look no further into our past than the COVID-19 pandemic for additional examples of communication mistakes that may linger in their impact in the form of vaccine hesitancy, resistance to masking, or, more broadly, distrust in public health interventions (10, 11). Smith et al. went so far as to call on epidemiologists to step up and become, “trusted and transparent communicators of their research findings” during the pandemic (12).

Some may argue that communication is not in the epidemiologic wheelhouse and should be left to communication specialists. Counterpoints to this perspective are that (1) published epidemiology competencies developed through varying processes with diverse stakeholders have identified communication repeatedly as a core competency; (2) epidemiologists are the experts on the epidemiological evidence and direct communication to the general public avoids the risk of poor translation through a communications intermediary, and finally (3) the media and the public will come to us regardless of whether we are prepared or not. Hence, the ability to communicate concisely, fairly, ethically and humbly appears to be a requisite skill for epidemiologists working in all sectors.

In this article, we summarize the literature supporting communication as a core competency for epidemiologists. We also discuss what skills under the broad communication umbrella are most important for epidemiologists facing the health challenges of the future and what communication training integrated into Masters and Doctoral programs might look like.

## The case for a core communication competency in epidemiologic training

The literature on epidemiology education and curriculum has long highlighted communication as an important skill domain. In 2002, the Association of Schools of Public Health (ASPH) held a workshop on doctoral education in epidemiology (13). The report described a communication competency domain that included communicating research results orally and in writing to scientists and non-scientists. The expectation was that doctoral students would have a facility with these skills rather than just a working knowledge of communication principles. The same year, the American College of Epidemiology (ACE) held an open forum on doctoral training in epidemiology (14). Participants of a discussion group concerning media skills felt that epidemiologists have an obligation to communicate their findings through the media and directly to the public. In 2015, Brownson et al. described the changing health communication environment as one of twelve macro trends to which epidemiologic training must be responsive (15). An assembled working group with representation from academia, practice and professional groups recommended more fully integrating training in epidemiology with training in communication and dissemination. Specifically, they recommended cultivating written and oral communication skills for multiple audiences, developing strategies to communicate uncertainty, improving data visualization skills, and teaching students how to harness communication technology tools including, mapping tools and social media.

In 2015 Huber Brunner et al. reported on results from two online surveys of 183 established epidemiologists in which respondents were asked to rate the importance of 19 domains and 66 competencies (16). Recent graduates from epidemiology Masters programs endorsed communication as an important skill domain for their current jobs and reported feeling inadequately prepared. A second article by Brownson et al. (17) focused on competencies needed for applied epidemiology jobs (17). Communicating epidemiologic findings effectively to health professionals, different decision-makers, and the public was listed as one of the five core purposes of applied epidemiology.

In 2018, the Department of Epidemiology at the Johns Hopkins Bloomberg School of Public Health hosted a symposium celebrating their centennial (18). Symposium participants, which included epidemiology department chairs, deans of schools of public health, epidemiology society leaders, editors of leading journals, and public health faculty, researchers, and trainees, discussed key areas of epidemiology curricula that the field should lean into to best prepare for emerging public health challenges. Communication was one of these areas. Symposium participants felt epidemiologists had a role as the “honest broker” of public health information and should be equipped with communication skills.

Werler et al. discussed gaps in epidemiology curricula that should be addressed to meet future challenges (19). The ability to effectively communicate was identified as one of the critical gaps, and the soft skill of oral communication was defined as the “ability to take the pulse of a group and adapt one’s message accordingly, including the content and style, to improve the exchange of information.” They labeled this skill as “emotionally intelligent communication.”

In 2020, the International Consortium on Teaching Epidemiology published a report on a multi-year effort to develop forward-thinking competencies for epidemiologists who pursue a career in an academic environment (20). Based on input from a diverse group of epidemiologists at various ranks from around the world, one of the five identified domains of competence was ‘communication and translation,’ which included competency G1: Competency to effectively communicate the results of health research to healthcare professionals, the lay public, and various media and thus contribute to debates concerning health and health care. The expected level of proficiency for this competency for a postdoctoral researcher in epidemiology working in an academic setting was 3 on a scale from 1 (basic knowledge) to 5 (proficiency), based on an online survey of the epidemiology community.

Hlaing et al. (21) reported on the results of the Doctoral Education in Epidemiology Survey, which collected information on doctoral-level competencies in accredited epidemiology doctoral degree programs in the United States and Canada (21). Competency to communicate with nonscientists was rated as either extremely or very important by 73% of respondents. The authors stressed the value of this competency, opining, “It is possible that epidemiologic findings are often limited in their impact because of our inability to effectively communicate study findings and articulate the value of our work to nonepidemiologists (e.g., heath care decision-makers, policymakers, the public).”

The endorsement of communication skills is consistent across much of the literature on training epidemiologists. Yet, as acknowledged by Lau et al., “typical curriculum does not emphasize effective communication sufficiently nor provide skills at understanding and tailoring our messages to various audiences” (18). It is fair to ask where is the disconnect? There are clear barriers to incorporating communication skills into epidemiology program curricula. First among those barriers is a lack of consensus on what skills under the large communication umbrella should be taught. Second is the challenge of training epidemiologists in skills that do not lend themselves well to a traditional classroom format; students will not learn most communication skills through lectures or typical classroom didactics. Third, the epidemiological discipline has evolved, and curricula have not caught up with the changing landscape of the field. Finally, there are few individuals within the field who have communication training who can both champion communication skill-building and develop the curriculum that would convey these skills.

In the next couple sections, we will address these barriers and provide guidance on how epidemiology graduate programs might move forward with building a meaningful communication competency into curriculum.

## Communication skills most needed by future epidemiologists

The 3rd International Meeting on Teaching Epidemiology took place in Zurich, Switzerland, on January 11–12, 2023, to discuss gaps in epidemiology training highlighted during the COVID-19 pandemic. One of the discussed gaps was communication. A survey before the meeting asked participants about communication competence: “What do you see as the specific set of skills that could be taught and assessed in a doctoral degree program?” The answers varied across a wide range of skills and ideas, including being able to communicate uncertainty, knowledge of communication theory/science, being able to confront misunderstanding/hostility, being able to write translation pieces/op-eds for the lay public, being able to translate numbers and terms into lay terminology, knowing how media works, interpersonal and listening skills, collaboration skills, storytelling skills, code-switching, being able to synthesize knowledge and debate skills. This heterogeneity in the vision of what communication skills look like for an epidemiology training program likely contributes to the lack of communication-focused curricula in epidemiology graduate programs. A better path forward is to identify the challenges we face and the communication skills that would best prepare epidemiologists to meet them.

Among those challenges, misinformation and disinformation have become an epidemic that will require an active response from the public health community. Misinformation is disproportionately targeted toward communities of color, who already have more reason to distrust the medical and research establishments due to experiences of discrimination and injustice (22). Epidemiologists working to counter mis-and disinformation will need training in skills that facilitate community engagement, particularly how to reach minoritized populations. Epidemiology graduate programs would need to provide opportunities for students to build confidence engaging these communities, training in and practice speaking to lay audiences with cultural humility, and facility in applying techniques to counter negative and false messages. Engaging communities means having a two-way dialog, so training in active listening and communicating risk and uncertainty will be critical.

Generally, concepts of risk and probability are difficult for the public to grasp. A survey of pedestrians in five metropolitan areas asking about the meaning of the phrase “30% chance of rain tomorrow” revealed that most of the lay public would interpret this probability statement as referring to either the amount of time during the day that it will rain or the amount of area in the region that will be affected (23). Nearly 30% of U.S. adults are considered to have low numeracy (24), which means they have difficulty accessing, using, and interpreting much of the statistical information that is the usual language of epidemiologists. Training in how to communicate risk to lay audiences with low health literacy and numeracy skills is essential for epidemiologists who will be engaging with the general public, and best practices have been suggested (25, 26).

Similarly, epidemiologists have struggled with communicating uncertainty, often choosing to avoid discussing uncertainty fearing it will reduce trust or perceptions of risk. However, avoiding communicating uncertainty erodes the public’s trust when uncertainty around an outcome or relationship leads to changing messages. The era of big data and Artificial Intelligence (AI) driven prediction tools has added new frontiers of uncertainty and an urgent need for skilled communication regarding the realistic clinical utility given issues of overfitting, lack of transparency and causality, and poor generalizability particularly to minority communities (27). Research is still sparse on how various communication strategies for communicating uncertainty impact trust and decision-making (28, 29). However, studies do suggest that graphical displays may help people recognize and understand uncertainty (30, 31), pointing to a need for data visualization and informational graphics skills.

Lastly, digital engagement may be as important as face-to-face engagement, given the broad reach of social media and the need for content that can counter viral misinformation. A few epidemiologists have leaned into social media as an integral part of their research. Skills that might serve researchers who want to develop a social media presence include story-telling, translating science for a general audience, cultural humility, and collaboration skills (32).

### Addressing barriers to adding communication training as a key feature of epidemiologic graduate curriculum

First among barriers is a lack of consensus on what communication skills should be taught. Another way to see this barrier is as a disconnect between the communication training that students actually receive, and the communication skills demanded by our current and future public health challenges. In a study that compiled epidemiology competencies from 48 accredited epidemiology PhD programs, data submitted to the Council on Education for Public Health (CEPH) suggested that 94% of programs listed a competency related to communication skills, which would seem to suggest programs are already focused on this set of skills (33). The disconnect is that many programs interpret communication competency as the ability to communicate with scientific peers, emphasizing formal scientific presentations and scientific manuscript development.

The ability to communicate effectively with the general public is the skillset emphasized in the literature and called for by current challenges. Skills under the broad communication umbrella that would prepare students to become honest brokers of public health information for the public would include:

- Public health communication planning
- Frameworks for advocating and influencing public health and health behavior
- Translating public health data for diverse levels of health literacy and numeracy
- Communicating risk and uncertainty
- How to evaluate your message impact on target audiences’ behaviors
- Using story-telling and anecdote to convey a message
- Communicating through visual media including data visualization
- Social media and digital engagement
- Strategies to counter misinformation and disinformation
- Cultural awareness, sensitivity and humility
- How to be an active listener

This list of skills represents a baseline for what would be needed by epidemiologists to effectively engage with the public and counter the growing epidemic of public distrust and misinformation. However, many additional skills would be useful for students including how to talk to the media, how to build partnerships with the community, written communication skills aimed at general audiences (op-eds or blogs) and advocacy skills, just to name a few. Programs could each tailor their communication focus to build an epidemiology workforce with a broad range of communication strengths.

The second challenge is that most communication skills will not be learned in a traditional classroom format. Coursework would need to be paired with practice through workshops that provided both simulated experiences (guests or faculty who play roles to allow students to experience various situations) or opportunities to practice in front of guests from the community who could provide feedback. These workshop experiences are critical, as communication can only be learned from opportunities to communicate. The Alda method, named after the actor Alan Alda, is science communication training that combines improvisational theater-based techniques with message-design strategies, recognizing that, while the message is important, connecting personally and emotionally with an audience is the key to building trust and shared empathy (34). Internships and practicum experiences, embedding students in media outlets or partnering with journalism or communication programs to work on communications projects, would give students real-world experience and insight into communication planning and implementation. Practicum experiences are used in most MPH and DrPH programs to give students real-world public health leadership and program-building experiences. Communication skills demand the same on-the-ground experiences to give context and meaning to classroom learning.

Third, the epidemiological discipline has evolved. More sophisticated methods of data analysis and technological tools like large language models and AI are increasingly part of the daily tool set at the same time the complexity of our health problems have demanded a broader and interdisciplinary set of skills. Graduate programs with limited credit-hour requirements have increasingly focused on training students in newer epidemiological and biostatistical methods, neglecting skills that fall outside the typical epidemiological wheelhouse. Curricula will need to catch up with the changing landscape through creative curricular approaches that weave in opportunities for building communication, collaboration and critical thinking skills (examples amongst a list of key skills needed by future epidemiologists) with conventional training in epidemiology.

Finally, who will champion curricular reform to ensure students acquire the communication skills they need to meet today’s and tomorrow’s challenges, and who will teach courses and lead workshops, given that most epidemiologists do not have these skills currently? Being a champion for this reform does not require expertise in communication but simply recognition that this skill set should now be considered critical for epidemiologists, as has been amply discussed in the literature. However, epidemiology departments will have to partner with science communication experts, journalism schools, media departments, and others to build a communication-focused curriculum. Those partnerships, however, could have benefits beyond building this competency in the public health workforce. It will take a village to counter health misinformation and rebuild trust. This is an opportunity to reach out to communities and bring them into the classroom to teach students how to engage with the public. Who better to prepare our students to communicate with the public we serve than the public?

## Discussion

The field of epidemiology has been beating the drum of communication competency for over two decades. Yet, we are still not training epidemiology students to communicate beyond the narrow slice of the population who are fellow epidemiologists. While there has not been a clear consensus on what skills under the communication umbrella should be taught, there is agreement that our audience is the general public with a particular focus on vulnerable communities most at risk of being targets for misinformation. Knowing our audience and our public health challenges highlights many of the specific skills we will need, namely the communication skills that would prepare students to become honest brokers of public health information for the public.

Of course curriculum change is a process that takes time, but given the clear and pressing need for epidemiologists to counter the epidemic of mis-and dis-information and rebuild public trust, at least incremental steps toward building a communication-savvy workforce should be taken now. A first step may be to bring together stakeholders—trainees, educators, program directors of epidemiology in academic settings, and practicing epidemiologists in government, for-profit, and non-profit organizations—to reimagine epidemiology curriculum, threading opportunities to engage and communicate with the public into programs. Our epidemiology organizations (Society for Epidemiologic Research, American College of Epidemiology) and Academic Public Health (i.e., Association of Schools and Programs of Public Health [ASPPH] representing schools and programs accredited by the Council on Education for Public Health [CEPH]) could be key drivers in such an effort.

By no means does there need to be a one-size-fits-all communication-focused curriculum. Epidemiology graduate programs may tailor the communication skill set to best equip students for particular communication tasks and contexts, building specific strengths. Diversity of communication skills across the field will ensure that collectively, we can rise to the challenges ahead and engage in myriad ways with diverse communities. Partnering with communication, media, and journalism experts as well as community members in building and implementing our communication-focused curricula could have benefits beyond the training of students, building collaborations that can fight the epidemic of mis-and disinformation.

Our curricula must train epidemiologists to be more than solid methodologists and astute thinkers; they must also be effective and competent communicators.

## Data Availability

The original contributions presented in the study are included in the article, further inquiries can be directed to the corresponding author.
